# Sleep Apnea and Cognitive Function in Heart Failure

**DOI:** 10.1155/2012/402079

**Published:** 2012-06-14

**Authors:** Krysten M. Knecht, Michael L. Alosco, Mary Beth Spitznagel, Ronald Cohen, Naftali Raz, Lawrence Sweet, Lisa H. Colbert, Richard Josephson, Joel Hughes, Jim Rosneck, John Gunstad

**Affiliations:** ^1^Department of Psychology, Kent State University, Kent, OH 44242, USA; ^2^Department of Psychiatry, Summa Health System, Akron, OH 44304, USA; ^3^Institute for Brain Sciences, Brown University, Providence, RI 02912, USA; ^4^Institute of Gerontology, Wayne State University, Detroit, MI 48201, USA; ^5^Institute on Aging, University of Wisconsin—Madison, Madison, WI 53706, USA; ^6^Harrington Heart & Vascular Institute, University Hospitals Case Medical Center and Department of Medicine, Case Western Reserve University School of Medicine, Cleveland, OH 44106, USA

## Abstract

*Background*. Prior research indicates that heart failure (HF) patients exhibit significant cognitive deficits on neuropsychological testing. Sleep apnea is associated with both HF and reduced cognitive function, but the combined impact of these conditions on cognitive function is unknown. *Methods*. In the current study, 172 older adults with a dual diagnosis of HF and sleep apnea or HF alone completed a battery of cognitive tests measuring attention, executive functioning, and memory. *Results*. Relative to patients with HF alone, persons with both HF and sleep apnea performed worse on measures of attention after adjusting for demographic and medical variables. *Conclusions*. The current findings suggest that HF patients with comorbid sleep apnea may be at greater risk for cognitive impairment relative to HF patient without such history. Further work is needed to clarify mechanisms for these findings and to determine whether the interactive effects on cognitive function lead to poorer patient outcomes.

## 1. Introduction

The American Heart Association estimates that heart failure (HF) affects more than five million adults and costs an estimated $30 billion annually in the United States alone [1]. As the population of older adults and the frequency of HF risk factors (e.g., hypertension and obesity) continue to rise, it is estimated that one in five adults will develop HF during their lifetime [1]. While the prevalence of this disease is alarming, so are the consequences—HF is a leading cause of hospitalization, morbidity, and mortality in the US [2–4].

HF is also a known risk factor for neurological disorders including Alzheimer's disease, stroke, and vascular dementia [5–7]. However, it is now known that cognitive deficits begin to manifest long before patients are diagnosed with these more serious conditions. For example, recent studies have suggested that up to 75% of persons with HF exhibit deficits on testing, including reduced performance on tests of memory, attention, executive function, and language [8–11].

A growing number of contributors to cognitive impairment in persons with HF have been identified, including structural brain changes, reduced cerebral blood flow, and autonomic nervous system disruption [12–16]. Although not previously examined, it appears likely that sleep apnea is another important contributor. Sleep apnea is common in persons with HF and has an independent adverse impact on cognitive function [17–21]. Recent work has shown that persons with sleep apnea score poorly on measures of attention [20–23], memory retrieval [24, 25], and executive functioning [17, 19, 23, 26]. Consistent with these cognitive deficits, persons with sleep apnea also exhibit hippocampal atrophy and decreased cerebral blood flow [27, 28].

Despite these findings, no study has examined the possible additive effects of sleep apnea on cognitive function in persons with HF. Based on the independent effects of sleep apnea in past studies, we hypothesized that patients with both disorders will demonstrate larger cognitive deficits than patients diagnosed only with HF.

## 2. Method

### 2.1. Participants

The local human subjects protection board approved the study protocol, and all participants provided written informed consent prior to participation.

 Participants were consecutively enrolled in a longitudinal study examining the neuropsychological aspects of HF. Strict inclusion/exclusion criteria were chosen for entry into the NIH-funded study to maximize generalizability to other samples and to capture the independent contribution of HF on cognitive function. Thus, participants of the current study met all criteria for parent study entry. Researchers recruited eligible participants from outpatient cardiology clinics. Inclusion criteria included English-speaking participants between 50 and 85 years of age with a documented history of HF. Exclusion criteria included a history of neurological disorder (e.g., stroke, Alzheimer's Disease, and severe head injury), history of significant psychological problems (e.g., schizophrenia, bipolar disorder, and substance abuse), or developmental disability (e.g., mental retardation).

### 2.2. Measures

#### 2.2.1. Neuropsychological Test Battery

Participants completed a battery of well-established neuropsychological measures assessing multiple domains. Researchers examined global cognitive functioning and cognitive performance in the domains of memory, language, and attention/executive function. An estimate of premorbid intelligence was also calculated. Participants completed the following measures:

**Global Cognitive Functioning**

*Modified Minimental Status Examination (3MS) [29]*. This test provides a screening measure of global cognitive function. It is comprised of several short tasks, including orientation, similarities, animal fluency, learning as well as brief and delayed recall of a short list of target words, and a copy of a simple geometric figure.

**Attention/Executive Function**

*Trail Making Test A and B [30].* In the Trail Making A task, participants connect a series of 25 numbered dots in ascending order as quickly as they can (e.g., 1-2-3, etc.). Trail Making B adds a set-shifting component and requires participants to alternate between numbers and letters in ascending order (e.g., 1-A-2-B, etc.).

*Frontal Assessment Battery [31]*. This test employs several short tasks to assess frontal system executive function. More specifically, participants identify similarities among words (e.g., automobile and boat), name as many words as they can that start with a target letter (e.g., words that begin with “M”), complete frontal-motor hand movements, and using the dominant hand, tap patterns requiring cognitive inhibition.

*Letter Number Sequencing [32]*.This test is a measure of complex attention and working memory. Researchers read strings of numbers and letters of increasing length, and participants reorganize the numbers and letters according to predetermined rules.

*Stroop Test [33]*. This test measures selective attention and mental flexibility. Participants first read columns of words spelling out colors printed in black ink (word subtest), then identify the color of the ink in which a series of *X*'s is printed (color subtest) and finally indicate the ink color of a word (which spells out a color), ignoring the verbal content (color-word subtest). Researchers calculated an interference score based on word and color subtest performances to determine expected performance on the color-word subtest and compared the score to actual color-word test performance.

*Digit Symbol Coding [34]*. This speeded task requires individuals to code numbers based on the corresponding symbols. It is a valid and reliable measure of visuomotor speed and complex attention.

**Memory**

*California Verbal Learning Test-Second Edition (CVLT) [35]*. Individuals learn, recall, and recognize a 16-item word list. Specific variables include indices of learning (sum of trials 1–5), immediate recall, delayed recall, and recognition.

### 2.3. Cardiovascular Measures

**Cardiovascular Fitness**

Researchers assessed cardiovascular endurance, used as a proxy for HF severity, with a 2-minute step test [36]. Participants marched in place for two minutes, bringing each knee up to a marked target on the wall set individually at the midpoint between each participant's hip and knee. The number of times the right knee met this point was counted.

### 2.4. Depressive Symptoms

**Beck Depression Inventory II (BDI-II) [37]**

Individuals complete a 21-item self-report measure to evaluate the presence and severity of depression. This measure has good psychometric properties. Higher scores on the BDI-II indicate more severe depressive symptoms.

### 2.5. Demographic and Medical History

History of sleep apnea, along with other medical and demographic characteristics, was collected through a review of participants' medical charts and self-report. Specifically, a medical record review was conducted for all participants to corroborate self-report and to ascertain a physician diagnosis of sleep apnea.

### 2.6. Analyses

All cognitive measures comprising each domain were converted to standardized *T* or *z* scores using published normative data. Multivariate analysis of covariance (MANCOVA) was then performed with neuropsychological measures of attention, executive function, and memory as dependent variables. Current medical history of sleep apnea (1 = positive history, 0 = negative history) served as categorical independent variable in each analysis. Demographic and medical covariates included education, heart failure severity (as estimated by the two minute step test), depressive symptomatology (as assessed by the BDI-II), and history of type 2 diabetes and hypertension (1 = positive history, 0 = negative history) were entered as covariates.

## 3. Results

### 3.1. Cognitive Impairment Is Prevalent in the Current Sample of Older Adults with HF

The overall sample had an average 3MS score of 92.69 ± 5.49. Within the sample, 29.4% had a 3MS score below 90, 34.5% between 90 and 95, and 36.1% between 95 and 100. HF patients with sleep apnea performed significantly worse on the 3MS than HF patients with no such history (*t*(178) = 7.57, *P* < .05). Specifically, HF patients with no history of sleep apnea (*N* = 138) had an average 3MS score of 93.32 ± 4.95, while HF patients with sleep apnea (*N* = 42) averaged 90.62 ± 6.64 on the 3MS.

### 3.2. Demographic and Medical Differences between HF Patients with and without Sleep Apnea

The original sample consisted of 180 enrolled persons with HF. As a result of missing data, 8 participants were excluded yielding a final sample size of 172 participants. The excluded participants did not differ in age (*t*(178) = −.391, *P* = .70), gender (*χ* ^2^(1, *N* = 180) = .45, *P* = .50), education (*t*(178) = .99, *P* = .33), 2-minute step test (*t*(178) = .75, *P* = .45), the BDI-II (*t*(178) = −.26, *P* = .80), history of diabetes (*χ* ^2^(1, *N* = 180) = 2.53, *P* = .11), or history of hypertension (*χ* ^2^(1, *N* = 180) = 1.60, *P* = .21).

 After exclusion of missing data, the current analyses included 172 older adults (132 HF patients, *M* = 68.6 years, 35% female; 40 HF and sleep apnea patients, *M* = 67.1 years, 42.5% female). Sleep apnea was common in the current samples of HF patients, with 23.3% having a history according to medical records. Independent samples *t*-test and chi square statistics were conducted to identify possible differences between HF patients with and without sleep apnea on important demographic and medical variables. No significant between-group differences were found for age, gender, or education. However, HF patients with sleep apnea performed significantly worse on the two-minute step test and were more likely to have hypertension and diabetes. See Table 1 for complete demographic and clinical characteristics. 

### 3.3. Sleep Apnea and Cognitive Function in the Current Sample of Older Adults with HF

After adjusting for demographic and medical variables, MANCOVA revealed significant differences between patients with and without sleep apnea on measures of attention (Λ = .934, *F*(3,163) = 3.866, *P* = .011). See Table 2. Specifically, posttests showed that persons with sleep apnea had significantly reduced performance on Digit Symbol coding. HF patients with sleep apnea also performed worse on Trail Making Test A and Letter Number Sequencing, though differences were not significant at *P* < .05.

Although the omnibus test was not statistically significant for the effects of sleep apnea on measures of executive function (Λ = .970, *F*(3,163) = 1.653, *P* = .18), posttests revealed that patients with sleep apnea performed significantly worse on the Frontal Assessment Battery (*P* = .03). No between-group differences emerged on the memory tests (Λ = .966, *F*(4,162) = 1.417, *P* = .23).

## 4. Discussion

In the present study, patients diagnosed with both HF and sleep apnea exhibited poorer cognitive functioning than patients with HF alone, even after adjusting for key demographic and medical factors known to influence cognitive function in this population. Specifically, patients with both diagnoses demonstrated poorer attention test performance than patients with HF alone. This pattern is consistent with past literature, as both HF [8–11] and sleep apnea [17–21] are independently associated with reduced cognitive functioning. Several aspects of these findings warrant brief discussion.

Patients with HF and sleep apnea scored significantly lower than those with HF alone on measures of attention, including tests commonly used to measure attentional deficits in both populations [8–11, 21]. These findings are consistent with both the HF [8–11] and sleep apnea [21] literature that suggests that patients exhibit impairment on attention tasks. These results are also consistent with both the sleep apnea and HF-neuroimaging literature. HF patients commonly exhibit reduced grey matter volume in regions important for attention and executive functioning including the frontal cortex, hippocampus, and cingulate [38]. Cerebral atrophy and infarcts [12, 16, 39] as well as white matter hyperintensities [40, 41] are also common in this population. While imaging research in sleep apnea is mixed, studies have found significant grey matter loss in the frontal cortex, parietal cortex, temporal lobe, and cingulate as well as atrophy in hippocampal regions [28]. Future imaging research is needed to determine the possible interaction of HF and sleep apnea on neuroimaging indices, as it may help clarify the mechanisms by which these disorders adversely impact the brain.

Clinically, these findings are important for both patient populations. Previous work indicates that HF patients with cognitive impairment are at elevated risk for mortality, re-hospitalization, and reduced functional independence [42–45]. The current findings suggest that persons with both HF and sleep apnea may be at elevated risk for such problems. Managing two diseases (e.g., medication regimen, scheduling, and attending appointments, adhering to physician instruction) requires more cognitive resources than one. If these findings are replicated, HF patients may benefit from regular discussion of possible sleep problems.

In contrast to the above, participants with both HF and sleep apnea did not exhibit poorer memory performance than those with HF alone. While memory dysfunction has been frequently found in HF patients [8, 9], contradictory findings exist in the sleep apnea literature. Short-term verbal [19, 24, 25] and visual [18, 46] memory impairments have been associated with sleep apnea in some studies, but not others [47, 48]. Similarly, studies examining procedural memory [24, 49] and long-term semantic memory [18, 46] have been equivocal [19, 23, 47]. It is also possible that the generally intact memory performance of the current sample (typical performance was within the average range) limits the statistical power of these analyses. Longitudinal studies are needed to determine whether memory function declines more rapidly in persons with both HF and sleep apnea. Given the elevated risk of Alzheimer's disease in persons with HF [5], prospective studies are needed to determine whether persons with both HF and sleep apnea are at elevated risk for future memory decline.

The current findings are limited in several ways. The current study investigates persons with HF and sleep apnea or HF alone but does not include a sleep apnea-only condition. Due to this limitation, we were not able to directly compare the cognitive effects of HF, sleep apnea, and HF plus sleep apnea in participants. Sleep apnea has been linked with abnormalities on neuroimaging (i.e., hippocampal atrophy and decreased cerebral blood flow) and deficits in cognitive function independent of HF [17–21, 27, 28]. Thus, while the current study suggests that comorbid sleep apnea is associated with additive deficits in cognitive function among HF patients, it remains unclear whether the interaction between sleep apnea and HF produces cognitive impairment beyond the pathophysiological effects of sleep apnea alone. Comparing test performance in a demographically matched group of patients with sleep apnea would provide a better understanding of the contribution of this condition to cognitive impairments in persons with HF and allow researchers to determine if the effect is additive or interactive. Additionally, as noted above, prospective studies are needed to determine whether the combination of HF and sleep apnea produce differential patterns of cognitive decline. Past work shows that each is associated with greater risk for Alzheimer's disease [5] though little is known about their possible interaction.

Another limitation of the current analyses involves limited information about the severity of HF and sleep apnea. Although we statistically controlled for acute HF severity in the primary analyses, it is possible that persons with both HF and sleep apnea differ on important factors that were unavailable to us, such as duration of HF and lower ejection fraction, among others [50]. It seems particularly worthwhile for future studies to examine the contribution of depression on cognitive function in these populations, as depression is prevalent in both HF and sleep apnea populations [51–54] and has been independently linked with cognitive impairment [17–21]. Similarly, cognitive function in sleep apnea populations has been suggested to decline as sleep apnea severity increases [21]. The current study assessed diagnostic history of sleep apnea and future studies should assess sleep apnea severity using more objective measures of sleep measures (i.e., polysomnogram sleep study) to elucidate the additive effects of sleep apnea on cognitive function in persons with HF. Finally, participants did not complete a sleep study as part of the current protocol, and categories were based on examination of medical records and self-report. Given the high prevalence rates, it is possible that some participants had undetected sleep apnea, thus limiting the observed findings. Future work is needed to clarify this possibility.

In summary, the current findings indicate that patients diagnosed with both HF and sleep apnea exhibit greater cognitive dysfunction than patients with HF alone. Future studies are needed to clarify the mechanisms for these patterns, as well as their clinical implications.

## Figures and Tables

**Table 1:** Demographic and clinical characteristics of 180 older adults with heart failure with and without sleep apnea.

Demographic Characteristics	HF patients	HF patients		
	with SA	without SA	*χ* ^2^(*p*)	*t*(*p*)
	(*N* = 42)	(*N* = 138)		
Age, mean (SD)	66.90 (8.43)	68.55 (11.00)	—	.89 (.37)
Female (%)	40.48	34.78	.45(.50)	—
Education, mean (SD)	12.93 (2.10)	13.51 (3.13)	—	1.39 (.17)
Clinical characteristics				
Diabetes (%)	52.38	31.16	6.29 (.01)	—
Hypertension (%)	88.10	64.50	8.54 (.003)	—
2MST, mean (SD)	49.76 (23.75)	63.45 (23.22)	—	3.33 (.001)

*Note. *2MST: 2-minute step test.

**Table 2:** Means and standard deviations (means (SD)) of cognitive tests for heart failure/patients with and without sleep apnea.

	*N*	Attention			Executive function			Memory			
		TMT-A^a^	DigistSym**	LNS	TMT-B**	FAB^a^	Stroop**	SDFR	SDCR	LDFR	LDCR
HF w/o SA	138	.082 (1.06)	9.63 (2.72)	10.15 (2.52)	−.398 (1.53)	−.448 (1.98)	45.46 (10.77)	−.210 (1.04)	−.243 (1.03)	−.315 (.96)	−.257 (.96)
HF w/SA	42	−.631 (1.42)	7.48 (2.23)	10.10 (3.33)	−1.62 (2.48)	−1.68 (2.50)	39.98 (8.06)	−.452 (1.11)	−.441 (1.14)	−.33 (1.32)	−.345 (1.17)

*Note.* ^a^ marginal significance; *P < .05; **P < .01.

Abbreviations: TMT-A: Trail Making Test A; Digitsym: Digit Symbol coding; LNS: letter number sequencing; TMT-B: Trail Making Test B; FAB: frontal assessment battery; Stroop: stroop interference effect; SDFR: California Verbal Learning short-delay free recall; SDCR: California Verbal Learning short-delay cued recall; LDFR: California Verbal Learning long-delay free recall; LDCR: California Verbal Learning long-delay cued recall.
